# Impact of prior JAK-inhibitor therapy with ruxolitinib on outcome after allogeneic hematopoietic stem cell transplantation for myelofibrosis: a study of the CMWP of EBMT

**DOI:** 10.1038/s41375-021-01276-4

**Published:** 2021-05-22

**Authors:** Nicolaus Kröger, Giulia Sbianchi, Tiarlan Sirait, Christine Wolschke, Dietrich Beelen, Jakob Passweg, Marie Robin, Radovan Vrhovac, Grzegorz Helbig, Katja Sockel, Eibhlin Conneally, Marie Thérèse Rubio, Yves Beguin, Jürgen Finke, Paolo Bernasconi, Elena Morozova, Johannes Clausen, Peter von dem Borne, Nicolaas Schaap, Wilfried Schroyens, Francesca Patriarca, Nicola Di Renzo, Zeynep Arzu Yeğin, Patrick Hayden, Donal McLornan, Ibrahim Yakoub-Agha

**Affiliations:** 1 Department of Stem Cell Transplantation University Medical Center Hamburg-Eppendorf, Hamburg, Germany; 2 Department of Biology, University of Rome “Tor Vergata”, Rome, Italy; 3 EBMT Data Office, Leiden, Netherlands; 4 Essen University Hospital, Essen, Germany; 5 University Hospital of Basel, Basel, Switzerland; 6 Hopital St. Louis, Paris, France; 7 University Hospital Center Rebro, Zagreb, Croatia; 8 Silesian Medica Academy, Katowice, Poland; 9 University Hospital Dresden, Dresden, Germany; 10 Hope Directorate St. James’s Hospital, Dublin, Ireland; 11 Hopital d’Enfants, Vandoeuvre Nancy, France; 12 University of Liege and CHU of Liege, Liege, Belgium; 13 University of Freiburg, Freiburg, Germany; 14 IRCCS Policlinico San Matteo, Pavia, Italy; 15 First State Pavlov Medical University of St. Petersburg, St. Petersburg, Russia; 16 Ordensklinikum Linz - Elisabethinen, Linz, Austria; 17 Leiden University Hospital, Leiden, Netherlands; 18 Radboud University Medical Centre, Nijmegen, Netherlands; 19 Antwerp University Hospital (UZA), Antwerp Edegem, Belgium; 20 Division of Hematology and Stem Cell Transplantation Center, University Hospital and DAME, Udine, Italy; 21 Unita Operativa di Ematologia e Trapianto di Cellule Staminali, Lecce, Italy; 22 Gazi University Faculty of Medicine, Ankara, Turkey; 23 Department of Haematology, Trinity College Dublin, St. James’s Hospital, Dublin 8, Ireland; 24 Department of Haematology, Guy’s Hospital and Department of Stem Cell Transplantation, University College London Hospital, London, England; 25 CHU de Lille, Univ Lille, INSERM U1286, Infinite, Lille, France

**Keywords:** Translational research, Myeloproliferative disease

## Abstract

JAK1/2 inhibitor ruxolitinib (RUX) is approved in patients with myelofibrosis but the impact of pretreatment with RUX on outcome after allogeneic hematopoietic stem cell transplantation (HSCT) remains to be determined. We evaluated the impact of RUX on outcome in 551 myelofibrosis patients who received HSCT without (*n* = 274) or with (*n* = 277) RUX pretreatment. The overall leukocyte engraftment on day 45 was 92% and significantly higher in RUX responsive patients than those who had no or lost response to RUX (94% vs. 85%, *p* = 0.05). The 1-year non-relapse mortality was 22% without significant difference between the arms. In a multivariate analysis (MVA) RUX pretreated patients with ongoing spleen response at transplant had a significantly lower risk of relapse (8.1% vs. 19.1%; *p* = 0.04)] and better 2-year event-free survival (68.9% vs. 53.7%; *p* = 0.02) in comparison to patients without RUX pretreatment. For overall survival the only significant factors were age > 58 years (*p* = 0.03) and HLA mismatch donor (*p* = 0.001). RUX prior to HSCT did not negatively impact outcome after transplantation and patients with ongoing spleen response at time of transplantation had best outcome.

## Introduction

The BCR-ABL1-negative myeloproliferative neoplasms primary myelofibrosis (PMF) and advanced forms of essential thrombocythemia and polycythemia vera (i.e., post ET/PV myelofibrosis) are chronic hematological malignancies characterized by splenomegaly, leukoerythroblastosis, extramedullary hematopoiesis and constitutive mobilization of CD34-positive progenitor cells. Patients with symptomatic PMF have a median survival of <5 years [1].

Before the introduction of JAK-inhibitor ruxolitinib (RUX), conventional therapies for treatment of PMF/MF included the use of growth factors such as erythropoietin, androgens, immune-modulating drugs, interferon-alpha, cytoreductive agents, and non-pharmacological options such as blood transfusion, spleen irradiation, and splenectomy. None of these approaches have shown to prolong patient survival. Allogeneic stem cell transplantation (HSCT) is the only currently available therapy with curative potential for MF, resulting in resolution of bone marrow fibrosis, molecular remission, and restoration of normal hematopoiesis [2].

However, allo-SCT is associated with a significant mortality and the European Leukemia Network (ELN) recommends consideration of allo-SCT in patients with a life expectancy of <5 years (i.e., intermediate II and high risk according to IPSS) [3].

JAK2V617F mutation is an acquired point mutation in the pseudo-kinase domain of the Janus kinase-2, which confers a constitutive JAK2 pathway activation with resulting growth factor independent proliferation of myeloid precursors [4, 5].

Ruxolitinib (RUX) is the first JAK inhibitor approved by the U.S. Food and Drug Administration for use in patients with intermediate- or high-risk MF (primary MF, Post PV/ET-MF) and in Europe for symptomatic MF patients with splenomegaly, regardless of the IPSS risk classification. RUX, a JAK1/ JAK2 inhibitor, showed early and sustained clinical benefits in patients with intermediate-2 and high-risk MF, including spleen size reduction and improvement of constitutional symptoms in a phase 1/2 trial (INCB18424-251) and the phase 3 trials COMFORT-I and COMFORT-II independent on JAK mutation status [6–8]. A survival benefit with RUX was shown in the COMFORT-I and COMFORT-II analyses [9, 10].

However, JAK inhibition only marginally targets the malignant clone and thus cannot be considered as curative treatment. Because spleen size may have significant impact on engraftment and graft function [11] and constitutional symptoms are a major risk factor for mortality [12], JAK inhibitor treatment prior to HSCT may be a reasonable option to decrease spleen size and improve constitutional symptoms in order to reduce therapy-related complications after stem cell transplantation.

This large retrospective international registry study of the European Society of Blood and Marrow Transplantation (EBMT) aimed to analyze the impact of RUX treatment prior to HSCT on outcome such as engraftment, graft-versus-host disease (GVHD), non-relapse mortality (NRM), relapse and overall survival (OS) in comparison compared to patients who received in the same time period allo-SCT without RUX pretreatment.

## Patients and methods

### Study design

This is a retrospective study utilizing registry data of EBMT. A preceding survey was done to invite EBMT’s center members to participate in this study and to identify eligible patients. Related variables which already exists in EBMT registry was extracted in June 2018. Thereafter pre-filled electronic forms were built in Microsoft excel incorporating all essential variables and existing variables from EBMT’s registries when applicable. The electronic forms were sent to centers which previously confirmed participation in this study. Major research objectives were to evaluate the impact of pretreatment RUX on spleen size, engraftment, NRM, GVHD, relapse incidence (RI), 2-year event-free and OS.

Major inclusion criteria were: Patients with PMF or myelofibrosis post polycythemia vera or essential thrombocythemia, allogeneic hematopoietic stem cell transplantation (HSCT) from related or unrelated donor matched or mismatched donor between 2012 and 2016 with or without RUX treatment prior to transplantation, aged 18–75 years and written informed consent. We included only patients who did receive RUX prior to conditioning. Patients with RUX through the transplant or posttransplant were not included in this analysis

### Statistical methods

Baseline variables consist of information available at time of HSCT. Continuous variables are summarized by reporting the number of patients with available data, median, and range. Categorical information has been reported showing the number of patients with available data, frequencies and percentages. For the calculation of percentages, the denominator has been determined by the number of available cases of the respective variable. Mann–Whitney or Kruskal–Wallis test have been used to compare continuous predictors among RUX and non-RUX treated cases and Chi-Squared or Fisher Exact Test for categorical data.

Outcome variables consider information available only after HSCT. The study aims to compare the following outcomes post – HSCT:

Non-relapse mortality (NRM), defined as the time from transplant until death from any cause without prior relapse/progression occurrence; relapse is considered as competing event. Relapse incidence (RI), defined as the time from HSCT until the first relapse or progression. Death without prior relapse/progression is a competing event. Event-free survival (EFS), defined as the time from HSCT until relapse, disease progression, or death, whichever occurs first. Overall survival (OS), defined as the time from HSCT until death from any cause. Acute GVHD, defined as the time from transplant until acute GvHD occurrence by day 100; death without prior acute GVHD is the competing event. Chronic GVHD, defined as the time from transplant until chronic graft disease occurrence from day 100; death without prior chronic GVHD is the competing event. Cases still alive are censored at the time of last follow-up. Time to Engraftment was defined as median days to neutrophil (>1.0 × 10e9/L) and platelet (>20 × 10e9/L).

For OS and EFS, the Kaplan–Meier estimates have been produced and groups have been compared using the Log-Rank test. The Cox proportional hazards (PH) regression model has been used to investigate the role of continuous prognostic factors and to get adjusted hazard ratios. The above outcomes are reported and compared, where possible, at 24 months post-HSCT.

Spleen response was defined as at least 25% reduction in spleen size, <25% was defined as no response. Within the group of responders more or <50% were also distinguished.

For all other endpoints methods for competing risks have been applied. In particular, crude cumulative incidence curves have been produced, and groups have been compared by the Gray test. Adjusted analyses of the cause-specific hazards have been performed using the Cox PH model. The above outcomes are reported and compared, where possible, at 12 or 24 months post-HSCT, according to the study objective indication.

Factors showing a significant impact on the outcome or highly associated with RUX treatment have been included in the final model.

In order to take into account possibly unmeasured confounders related to the fact of belonging to the same center, adjusted effects on outcomes have been estimated in terms of hazard ratios using the Cox model with a shared random center effect (“Frailty Model”).

In order to further investigate the role of the different length of the time intervals between diagnosis and transplant in the two groups, RUX and no RUX cases, on survival like endpoints, adjusted hazard ratios for OS and EFS have been estimated using model for left truncated data. The triplet given by the transplant time, the survival since diagnosis and the endpoint specific status was taken as outcome variable. As a further step, Poisson regression models for multiple time scales have been applied to asses and evaluate the impact of both time scales, the interval time diagnosis and transplant and the follow up from transplant onwards. As these analyses have not provided any significant results, data are not shown.

All outcomes are reported either with a survival or cumulative incidence plot. Tables of survival and cumulative incidence estimates at the specified time points also report numbers of patients at risk and 95% confidence intervals (CI). 95% CI are also reported for hazard ratios from Cox regression. All comparisons are reported with an associated *p* value, and *p* values < 0.05 are considered statistically significant.

The quality of follow-up in the entire cohort has been determined using the reverse Kaplan–Meier method, providing the median follow-up time and associated 95% confidence interval.

The frequency of missing cases is displayed in the frequency table of the corresponding variable. These counts of missing cases have not been included in the calculation of any of the percentages, test statistics or subsequent *p* values. In case data were missing for a specific variable, the number of patients contributing to that variable have been lower than the number of patients in the respective cohort. Where necessary, in order to avoid reduction of sample size, cases with missing information have been included by adding the missing category for factors included in Cox model.

## Results

Fifty-eight EBMT centers participated in the study and completed the electronic forms. Of those centers, 586 patients’ data were received. Thirty-five patients were excluded due to inclusion of ongoing clinical trials, transformation to other malignancies before allo-SCT, having syngeneic donor and error diagnosis information entered in the registry. In total, 551 patients were included on which two hundred seventy-seven patients received RUX treatment prior to allo-SCT. The patient’s characteristics at study entry are listed in Table 1. In the RUX arm there were more intermediate II and fewer intermediate I patients and more JAK2-negative patients. MUD and Karnofsky score of ≤80 were more frequently seen in RUX pretreated patients. In addition the interval from diagnosis to transplantation was significantly longer in the RUX pretreated arm.

**Table 1:** Patients characteristics at study entry (*n* = 551).

	Prior RUX	No RUX	*p* = value
Number of patients	*n* = 277 (50.3%)	*n* = 274 (49.7%)	
Median age (range)	58 (30–75)	58 (29–75)	*p* = 0.4
Patients gender (*n* = 551)			
Male	*n* = 175 (63%)	*n* = 173 (63%)	*p* = 0.9
Female	*n* = 102 (37%)	*n* = 101 (37%)	
DIPSS at transplant (*n* = 421, 76%)			
Low	*n* = 2 (1%)	*n* = 11 (6%)	*p* < 0.01
Intermediate-1	*n* = 48 (21%)	*n* = 69 (35%)	
Intermediate-2	*n* = 125 (56%)	*n* = 76 (39%)	
High risk	*n* = 49 (22%)	*n* = 41 (20%)	
JAK (*n* = 354, 64%)			
Positive	*n* = 154 (79%)	*n* = 134 (86%)	*p* = 0.05
Negative	*n* = 44 (21%)	*n* = 22 (14%)	
Donor (*n* = 551, 100%)			
MRD	*n* = 66 (24%)	*n* = 100 (36%)	*p* = 0.003
MUD	*n* = 192 (69%)	*n* = 150 (55%)	
MMUD/MMRD	*n* = 19 (7%)	*n* = 26 (9%)	
CMV status (*n* = 533, 97%)			
+/+	*n* = 113 (41%)	*n* = 108 (41%)	*p* = 0.56
+/−	*n* = 46 (20%)	*n* = 55 (21%)	
−/−	*n* = 90 (33%)	*n* = 75 (29%)	
−/+	*n* = 23 (9%)	*n* = 23 (9%)	
Disease (*n* = 551, 100%)			
Primary myelofibrosis	*n* = 185 (67%)	*n* = 199 (73%)	*p* = 0.1
Post-ET/-PV	*n* = 92 (33%)	*n* = 75 (27%)	
Median follow-up (months)	44 (6–87)	49 (2–91)	*p* < 0.01
Conditioning regimen (*n* = 548, 99%)			
RIC	*n* = 187 (67%)	*n* = 164 (60%)	*p* = 0.08
MAC	*n* = 90 (33%)	*n* = 107 (40%)	
Spleen size at transplant (palpable in cm) (*n* = 305)	10 (1–30)	8 (1–30)	*p* = 0.4
Constitutional symptoms at transplant (*n* = 297, 55%)	*n* = 159 (68%)	*n* = 138 (61%)	*p* = 0.1
Donor source (*n* = 551, 100%)			
BM	*n* = 21 (8%)	*n* = 23 (7.6%)	*p* = 0.9
PB	*n* = 255 (91.6%)	*n* = 250 (91%)	
CB	*n* = 1 (0.4%)	*n* = 1 (0.4%)	
Karnofsky at transplant (*n* = 537, 97%)			
≤80	*n* = 113 (42%)	*n* = 89 (33%)	*p* = 0.03
≥90	*n* = 154 (58%)	*n* = 181 (67%)	
Interval from diagnosis to transplant (months)	68 (2–430)	32 (2–527)	*p* < 0.01

Out of 551 cases, 277 received RUX at any time prior to transplantation and 274 did not receive RUX. The major characteristics of the RUX pretreated cohort regarding treatment duration, dosing and response are listed in Table 2. The median spleen size at start of RUX was 12 cm below left costal arch and the median spleen size in the RUX treated group at time of transplantation was 10 cm while in the non-RUX group the median spleen size was 8 cm at time of transplantation.

**Table 2:** Ruxolitinib treatment prior to allograft (*n* = 277).

Discontinuation of RUX prior to allograft (for reasons other than transplant) (*n* = 245, 88%)	*n* = 56 (23%)
Tapering of RUX prior to discontinuation (*n* = 245, 88%)	
Yes	*n* = 117 (48%)
No	*n* = 128 (52%)
Median starting dose of RUX/day	30 mg (range: 5–80)
Median dose at last period/day	20 mg (range: 5–50)
Reasons of early discontinuation (*n* = 56, 100%)	
No response	*n* = 16 (29%)
Loss of response	*n* = 5 (9%)
Toxicity	*n* = 13 (23%)
Others	*n* = 22 (39%)
Rebound phenomenon after stopping RUX (*n* = 245, 88%)	*n* = 15 (6%)
Median spleen size at start of RUX (palpable in cm) (*n* = 141, 51%)	12 cm (1–32)
Constitutional symptoms at start of RUX (*n* = 219, 79%)	
Yes	*n* = 190 (87%)
No	*n* = 29 (13%)
Best response to RUX (*n* = 227, 82%)	
Spleen size > 50%	*n* = 39 (17%)
Spleen size < 50%	*n* = 88 (39%)
No response	*n* = 100 (44%)
Response of RUX to spleen size at time of transplant (*n* = 195, 70%)	
Spleen response > 50%	*n* = 25 (13%)
Spleen response < 50%	*n* = 66 (34%)
Lost spleen response	*n* = 23 (12%)
No spleen response	*n* = 81 (42%)
Median duration of RUX treatment (months) (*n* = 219, 79%)	7.6
Infections during RUX treatment (*n* = 277, 100%)	
Yes	*n* = 25 (11%)
No	*n* = 210 (89%)

In order to compare outcome results after allogeneic stem cell transplantation in more detail, the RUX pretreatment group was divided into ongoing spleen response (*n* = 91) with spleen response ≥ 50% (*n* = 25) and spleen response <50% (*n* = 66), or no ongoing spleen response (*n* = 104): either loss of spleen response (*n* = 23) or no spleen response at all (*n* = 81).

### Engraftment/graft failure

**Fig. 1:**
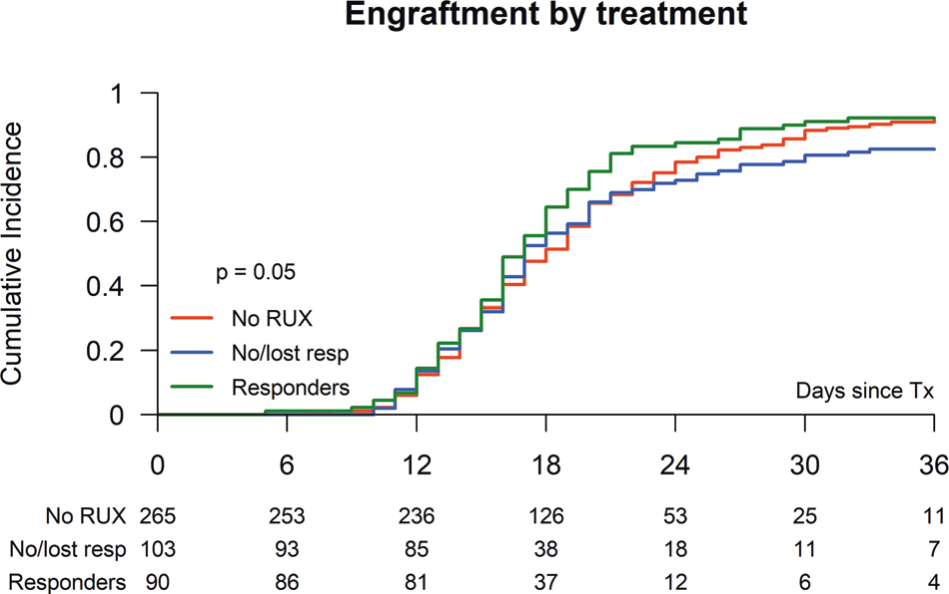
Engraftment after stem cell transplantation according Ruxo pretreatment. Neutrophil engraftment after allogeneic stem cell transplantation of ruxolitinib responder vs. no or lost responders vs. non-RUX pretreatment.

The median time to neutrophil engraftment for the entire study population was 17 days (range, 5–83) and for platelets 21 days (range, 3–413). For the non-RUXO treated patients the median neutrophil and platelet engraft was 17 (range 7–57) and 20 (range 3–413) days, respectively. For RUXO responsive patients the neutrophil and platelet engraftment was noted after a median of 16 (range 5–54) and 20 (range 6–395) days, while for patient who had no or lost response to RUXO the median time for neutrophil and platelet engraftment was 17 (range 10–81) (*p* = 0.43) and 25/range 5–198) days (*p* = 0.005), respectively. (Fig. 1 and Table 3)

**Table 3:** Results: univariate analysis.

	Overall (*n* = 551)	No RUX (*n* = 274)	No or lost response to RUX (*n* = 104)	RUX (response) (*n* = 91)	*p* = value
Non-relapse mortality (at 1 year)	21.9% (95% CI: 18–25)	22.9% (95% CI: 18–28)	25.5% (95% CI: 17–34)	14.8% (95% CI: 7–22)	0.16
Median days to neutrophil engraftment (range)	17 (5–83)	17 (7–57)	17 (10–81))	17 (5–83)	0.43
Acute GVHD II–IV	28.9% (95% CI: 25.1–32.8)	28.9% (95% CI: 23.4–34.4)	27.5% (95% CI: 18.8–36.1)	27.0% (95% CI: 17.7–36.2)	0.92
Chronic GVHD (at 2 years)	46.7% (95% CI: 42.2–51.2)	41.7% (95% CI: 35.4–48.0)	55.7% (95% CI: 45.2–66.2)	50.7% (95% CI: 40.0–61.3)	0.11
Chronic GVHD extensive disease	29.2% (95% CI: 24.7–33.6)	25.5% (95% CI: 19.8–31.1)	37.9% (95% CI: 27.5–48.3)	30.6% (95% CI: 20.8–40.4)	0.08
Overall survival at 2 years	63.2% (95% CI: 59.1–67.2)	60.8% (95% CI: 55.0–66.6)	57.5% (95% CI: 48.0–67.1)	70.0% (95% CI: 60.5–79.5)	0.15
Relapse at 2 years	16.3% (95% CI: 13.2–19.5)	19.1% (95% CI: 14.3–23.9)	15.7% (95% CI: 8.6–22.7)	8.1% (95% CI: 2.3–13.8)	0.05
Event-free survival at 2 years	56.5% (95% CI: 52.3–60.8)	53.7% (95% CI: 47.7–59.8)	49.9% (95% CI: 40.2–59.6)	68.9% (95% CI: 59.2–78.7)	0.01

### Graft-versus-host disease and CMV reactivation

The incidence of acute GVHD grade II–IV for the entire study population was 28.9% (95% CI: 25.1–32.8) and did not differ between the non-RUX (28.9%) (95% CI: 23.4–34.4) and RUX treatment (29.0%) (95% CI: 23.6–34.4) (*p* = 0.99), despite more patient in the RUX arm received unrelated donor grafts. The incidence of severe aGVHD II–IV did also not differ between, RUX-responsive (27.0%) (95% CI: 17.7–36.2) and no or lost response to RUX group (27.5%) (95% CI: 18.8–36.1) (*p* = 0.92).

The cumulative incidence of overall chronic GVHD at 2 years for the entire study population was 46.7% (95% CI: 42.2–51.2) and was significantly lower in the non-RUX arm (41.7%) (95% CI: 35.4–48.0) compared to the RUX arm (51.4%) (95% CI: 45.1–57.7) (*p* = 0.05). The incidence of cGVHD did not differ between RUX-responsive (50.7%) (95% CI: 40.0–61.3) and no or lost response to RUX group (55.7%) (95% CI: 45.2–66.2) (*p* = 0.11).

Chronic GVHD extensive disease was noted in 25.5% (95% CI: 19.8–31.1) in the non-RUX, 37.9% (95% CI: 27.5–48.3) in the no or lost response group, and 30.6% (95% CI: 20.8–40.4) in the RUX responsive group (*p* = 0.08) (Table 3).

Overall 31.7% experienced CMV reactivation after stem cell transplantation. Patients at risk (serostatus CMV positive) had a non-significant higher risk of CMV reactivation in the RUX pretreated arm (36.6% vs. 26.8%, *p* = 0.07).

### Non-relapse mortality (NRM)

The cumulative incidence of NRM at 1 year was 21.9% (95% CI: 18–25) and did not differ significantly in an univariate analysis between the non-RUX (22.9%) (95% CI: 18–28) vs. no or lost response to RUX (25.5%) (95% CI: 17–34) and the RUX responsive group (14.8%) (95% CI: 7–22) (*p* = 0.16) (Table 3). In a multivariate analysis (MVA) including RUX vs. non-RUX pretreatment the HR for RUX pretreatment was 0.80 (*p* = 0.32) (Table 4A), while ongoing spleen response vs. no/lost response showed a HR of 0.56 (*p* = 0.07). Higher age (>58 y) resulted in a HR of 1.46 (*p* = 0.07) while the only significant factor for a higher NRM in the MVA was HLA-mismatched donor (HR 2.79, *p* = 0.002) (Table 4A).

**Table 4:** A Multivariate analysis for non-relapse mortality. B Multivariate analysis for relapse incidence. C Multivariate analysis for event-free survival. D Multivariate analysis for overall survival.

Ruxolitinib pretreated yes vs. no			Ruxolitinib split according to the response		
Factor	HR (95% CI)	*p* value	Factor	HR (95% CI)	*p* value
RUX vs. No RUX	0.80 (0.51–1.25)	0.323	Ongoing vs. no/lost spleen response	0.56 (0.30–1.04)	0.069
Age: ≥58 vs. <58	1.40 (0.95–2.04)	0.086	Ongoing spleen response vs. No RUX	0.66 (0.36–1.20)	0.172
DIPSS: High vs. Other	1.13 (0.66–1.96)	0.651	No/lost response vs. No RUX	1.17 (0.62–2.20)	0.626
DIPSS: Missing vs. Other	1.19 (0.70–2.03)	0.525	Age: ≥58 vs <58	1.46 (0.97–2.21)	0.071
Unrelated vs. matched donor	1.73 (1.08–2.76)	0.023	DIPSS: High vs Other	1.08 (0.61–1.92)	0.781
Mismatched vs. matched donor	3.43 (1.86–6.32)	<0.01	DIPSS: Missing vs. Other	1.52 (0.85–2.72)	0.160
			Unrelated vs. matched donor	1.55 (0.95–2.52)	0.077
			Mismatched vs. matched donor	2.79 (1.45–5.36)	0.002
RUX vs. No RUX	0.68 (0.41–1.11)	0.121	Ongoing vs. no/lost spleen response	0.41 (0.15–1.09)	0.073
Age: ≥58 vs. <58	1.23 (0.83–1.83)	0.300	Ongoing spleen response vs. No RUX	0.34 (0.12–0.95)	0.039
DIPSS: High vs. Other	1.08 (0.73–1.58)	0.713	No/lost response vs. No RUX	0.83 (0.51–1.34)	0.449
DIPSS: Missing vs. Other	0.67 (0.40–1.12)	0.128	Age: ≥58 vs. <58	1.34 (0.91–1.96)	0.133
Unrelated vs. matched donor	0.80 (0.44–1.46)	0.467	DIPSS: High vs. Other	1.06 (0.66–1.70)	0.809
Mismatched vs. matched donor	0.65 (0.20–2.13)	0.474	DIPSS: Missing vs. Other	0.71 (0.38–1.31)	0.273
			Unrelated vs. matched donor	0.84 (0.42–1.66)	0.615
			Mismatched vs. matched donor	0.62 (0.16–2.44)	0.495
RUX vs. No RUX	0.81 (0.59–1.11)	0.196	Ongoing vs. no/lost spleen response	0.55 (0.37–0.81)	0.003
Age: ≥58 vs. <58	1.41 (1.10–1.80)	0.007	Ongoing spleen response vs. No RUX	0.61 (0.40–0.91)	0.016
DIPSS: High vs. Other	1.07 (0.74–1.53)	0.727	No/lost response vs. No RUX	1.11 (0.69–1.77)	0.676
DIPSS: Missing vs. Other	0.95 (0.69–1.29)	0.725	Age: ≥58 vs. <58	1.48 (1.12–1.97)	0.006
Unrelated vs. matched donor	1.18 (0.79–1.74)	0.422	DIPSS: High vs. Other	1.00 (0.70–1.44)	0.982
Mismatched vs. matched donor	1.94 (1.12–1.80)	0.017	DIPSS: Missing vs. Other	1.08 (0.79–1.47)	0.635
Interval Diagnosis-Transplant: +1 month	1.00 (1.00–1.00)	0.903	Unrelated vs. matched donor	1.16 (0.76–1.77)	0.493
			Mismatched vs. matched donor	1.87 (1.06–3.28)	0.030
			Interval Diagnosis-Transplant: +1 month	1.00 (1.00–1.00)	0.983
Ruxolitinib vs. No Ruxo	0.81 (0.59–1.13)	0.215	Ongoing vs. no/lost spleen response	0.69 (0.42–1.11)	0.123
Age: ≥58 vs. <58	1.37 (1.05–1.78)	0.021	Ongoing spleen response vs. No Ruxo	0.76 (0.50–1.17)	0.212
DIPSS: High vs. Other	1.19 (0.80–1.79)	0.391	No/lost response vs. No Ruxo	1.11 (0.67–1.85)	0.688
DIPSS: Missing vs. Other	1.10 (0.74–1.65)	0.629	Age: ≥58 vs. <58	1.42 (1.04–1.95)	0.026
Unrelated vs. matched donor	1.47 (1.02–2.12)	0.039	DIPSS: High vs. Other	1.16 (0.77–1.74)	0.485
Mismatched vs. matched donor	2.46 (1.45–4.19)	0.001	DIPSS: Missing vs. Other	1.27 (0.87–1.86)	0.216
Interval Diagnosis-Transplant: +1 month	1.00 (1.00–1.00)	0.536	Unrelated vs. matched donor	1.35 (0.92–2.00)	0.128
			Mismatched vs. matched donor	2.37 (1.40–4.03)	0.001
			Interval Diagnosis-Transplant: +1 month	1.00 (1.00–1.00)	0.464

### Relapse

**Fig. 2:**
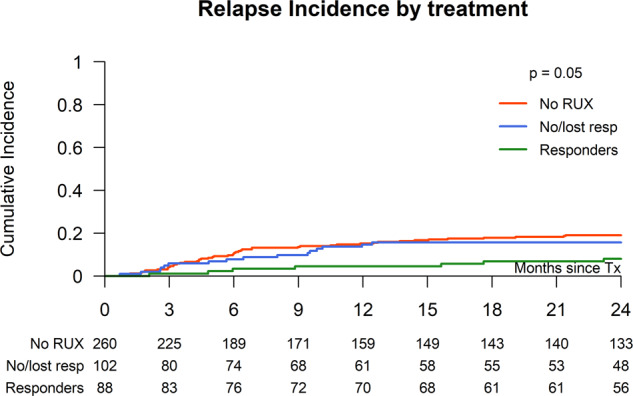
Relapse incidence after stem cell transplantation according Ruxo pretreatment. Cumulative incidence of relapse after allogeneic stem cell transplantation of RUX responder vs. no/lost responders vs. non-RUX pretreatment.

The cumulative incidence of relapse at 2 years for the entire study population was 16.3% and in patients with RUX pretreatment not significantly lower than in the non-RUX pretreated group: 13.7% (95% CI: 9.6–17.8) vs. 19.1% (95% CI: 14.3–23.9) (*p* = 0.09) (Fig. 2). The incidence of relapse at 2 years significantly differed between RUX responsive (8.1%; 95% CI: 2.3–13.8) patients and those who had no or lost response (15.7%; 95% CI: 8.6–22.7) (*p* = 0.05). In a MVA including RUX vs. non-RUX pretreatment the HR for RUX pretreatment was 0.68 (95% CI:0.41–1.11) (*p* = 0.12) (Table 4A), while ongoing spleen response vs. no/lost response showed a HR of 0.41 (95% CI: 0.15–1.09) (*p* = 0.07) and ongoing spleen response vs. non-RUX showed a HR of 0.34 (95% CI: 0.12–0.95, *p* = 0.04), which was the only significant factor in the MVA for relapse (Table 4B).

### Event-free survival

**Fig. 3:**
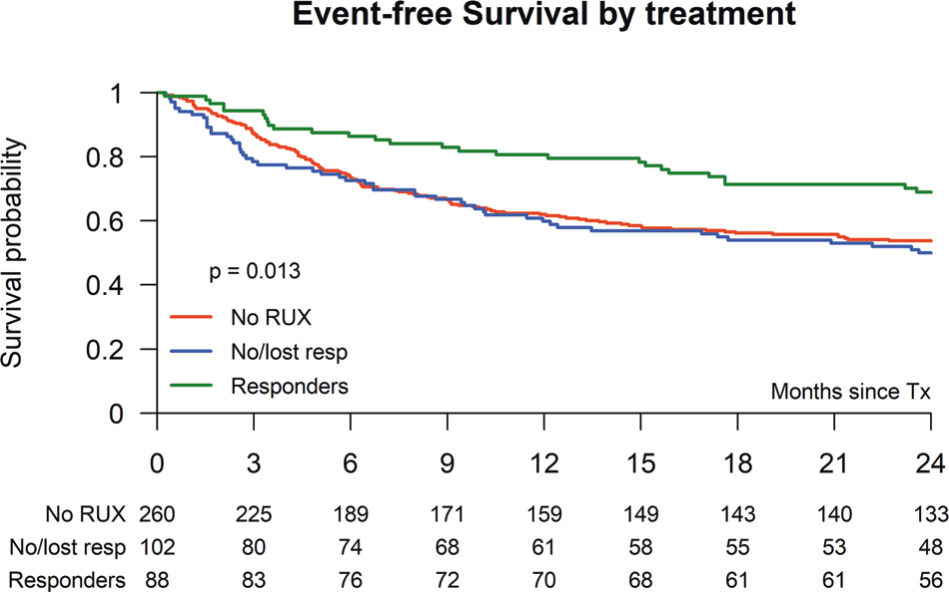
Event-free survival after stem cell transplantation according Ruxo pretreatment. Event-free survival after allogeneic stem cell transplantation of RUX responder vs. no/lost responders vs. non-RUX pretreatment.

The EFS for the entire study population at 2 years was 56.5% (95% CI: 52.3–60.8) and did not differ between RUX pretreatment or no pretreatment: 59.2% (95% CI: 53.3–65.1) vs. 53.7% (95% CI: 47.6–59.8) (*p* = 0.18). But EFS was significantly improved in RUX patients with ongoing spleen response (68.9%) (95% CI: 59.2–78.7) vs. those without RUX pretreatment (53.7%) (95% CI: 47.6–59.8) and those with no/lost response (49.9%) (95%CI: 40.2–59.6) (*p* = 0.01) (Table 3 and Fig. 3). In a MVA the HR of RUX vs. no-RUX pretreatment was 0.81 (95% CI: 0.59–1.11) (*p* = 0.19) (Table 4A), while ongoing spleen response vs. no/lost response showed a HR of 0.55 (95% CI: 0.37–0.81) (*p* = 0.003) and ongoing spleen response vs. non-RUX showed a HR of 0.61 (95% CI: 0.40–0.91) (*p* = 0.02). Other significant factors for EFS in the MVA were age >58 y (HR 1.48; 95% CI: 1.12–1.97, *p* = 0.006) and HLA-mismatched donor (HR 1.87; 95% CI: 1.06–3.28, *p* = 0.03) (Table 4C).

### Overall survival

**Fig. 4:**
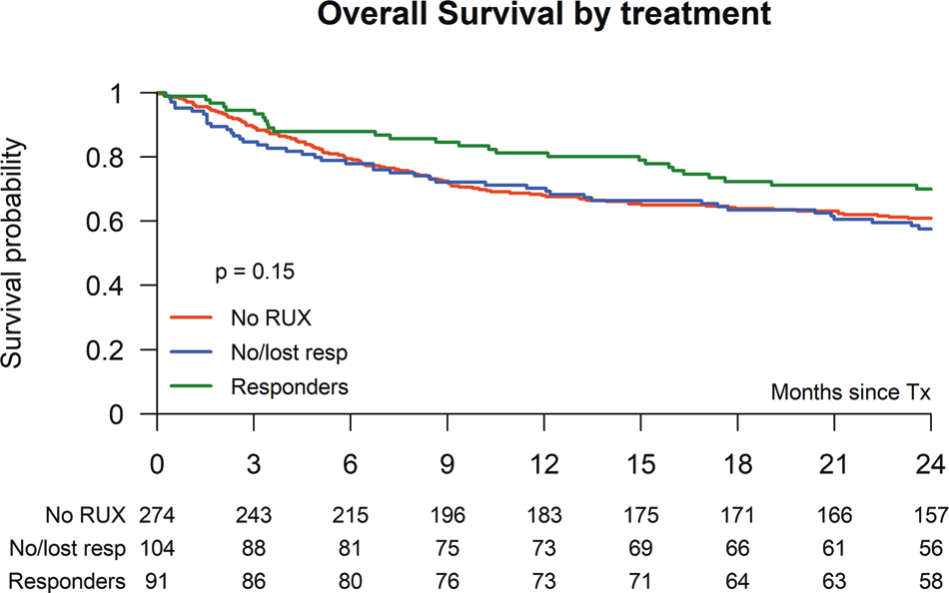
Overall survival after stem cell transplantation according Ruxo pretreatment. Overall survival after allogeneic stem cell transplantation of RUX responder vs. no/lost responders vs. non-RUX pretreatment.

The OS for the entire study population at 2 years was 63.2% (95% CI: 55.0–66.6) and did not differ between RUX pretreatment or no pretreatment: 65.5% (95% CI: 59.8–71.1) vs. 60.8% (95% CI: 55.0–66.6) (*p* = 0.22), and did also not differ in a univariate analysis between the no-RUX (60.8%, 95% CI: 55.0–66.6) vs. no/lost response to RUX (57.5%, 95% CI: 48.0–67.1) and the RUX responsive group (70.0%, 95% CI: 60.5–79.5) (*p* = 0.15) (Table 3 and Fig. 4). In a MVA for OS including RUX vs. non-RUX pretreatment the HR for RUX pretreatment was 0.81 (95% CI: 0.59–1.13) (*p* = 0.21) (Table 4D), while ongoing spleen response vs. no/lost response showed a HR of 0.69 (95% CI: 0.42–1.11) (*p* = 0.12) and ongoing spleen response vs. non-RUX showed a HR of 0.76 (95% CI: 0.50–1.17) (*p* = 0.21). The only significant factors in the MVA for OS were age >58 y (HR 1.42; 95% CI: 1.04–1.95, *p* = 0.03) and HLA-mismatched donor (HR 2.37; 95% CI: 1.40–4.03, *p* = 0.001) (Table 4D). It is of note that the interval between diagnosis and transplant did not influence survival.

## Discussion

The therapeutic effect of JAK-inhibition to reduce spleen size and improve constitutional symptoms and performance status is the rationale for using the drug pretransplant and improve outcome after HSCT for myelofibrosis. Several smaller retrospective and prospective studies investigated RUX as pretreatment prior to allogeneic stem cell transplantation with controversial results [13–20]. In two studies [13, 15] outcome after HSCT was particularly improved in patients with clinical improvement to RUX therapy while other reported side effects such as withdrawal symptom or an increased risk of infections [15, 16, 19], relative high incidence of graft failure [20] or in one prospective study tumor lysis syndrome, cardiogenic shock and sepsis [19]. The results of this large retrospective EBMT study confirmed safety and feasibility of using RUX prior to HSCT and supports the expert recommendation regarding the use of JAK-inhibitor RUX in the context of stem cell transplantation [21]. The majority of the centers followed the EBMT/ELN recommendations regarding discontinuation of RUX and only in 6% of the patients a rebound phenomenon after discontinuation of RUX were reported. However, none of these events were reported to be life threatening or required intensive treatment.

We observed a low risk of graft failure in patients who responded regarding spleen size to RUX prior to transplantation (6%) in comparison to RUX pretreated patients with no or lost spleen response prior to HSCT (15%), which highlights the role of spleen size regarding risk of graft failure in myelofibrosis patients [22]. This is further supported by 7% graft failure incidence of the non-RUX pretreated patients who had only a median spleen size of 8 cm at time of HSCT.

The presented large study which included 551 patients confirmed higher age and mismatched unrelated donors as significant factors for worse outcome, as shown by several prospective and retrospective studies [23–30]. In general the hazard ratio in RUX pretreated patients for NRM, relapse, EFS, and OS was always <1.0 ranging from 0.68 to 0.81, but did not reach statistical significance for these outcome variables. However, if the RUX pretreated patients were divided in patients with ongoing spleen response at time of transplantation and no or lost spleen response a clear benefit could be seen in a lower relapse rate which resulted in an improved EFS for patients who underwent HSCT with ongoing spleen response during RUX therapy. Furthermore, even if not significant (*p* = 0.07), patients with ongoing spleen response had a lower NRM rate with only 15% and an HR of 0.56 in MVA, further supporting the recommendation to transplant myelofibrosis patients with ongoing spleen response during RUX therapy rather than waiting until JAK inhibition treatment has failed. However, reduced relapse rate, lower NRM, and improved EFS did not translate into a significantly improved OS at 2 years, most likely because relapse can either be salvaged by donor lymphocyte infusion and/or second allograft, and a larger follow-up is needed for valid conclusion regarding differences in OS [31, 32]. An immunosuppressive effect of RUX has been described and affects mainly dendritic cells and T-cell populations [13, 33]. The drug has been investigated in steroid refractory acute GVHD and has shown higher response rate at day 28 in a randomized comparison to best available therapy [34]. In a smaller pilot study given RUX during transplant period a low incidence of acute GVHD has been described [35]. In our study no effect of RUX on the incidence of acute GVHD could be seen, most likely because of the short half-life of the drug which has been discontinued many days prior to graft infusion. The observed non-significant higher incidence of chronic GVHD in the RUX pretreated group might be best explained by the higher number of unrelated donor transplantation in the RUX arm.

Due to immunosuppressive effect some studies observed a higher incidence of CMV reactivation after HSCT in patients who received RUX prior to transplant and it is of interest that also in our study a higher but not significant incidence of CMV reactivation (36.6% vs. 26.8%) was observed in the RUX-pretreated CMV seropositive patients [15].

In our study we observed different lengths of time interval between diagnosis and transplantation in both arms with a considerable longer interval in the RUX pretreated arm, which is in line with a recent real world report on RUX treatment showing prolonged treatment even in patients with unstable or no spleen response. This suggested a delaying transplant strategy rather than a bridging strategy [36]. However, by using several statistical methods including left truncation or Poisson model we could not confirm that the variable time from diagnosis to transplant had any impact on event-free or OS in our study population. Although if other JAK inhibitors like fedratinib, pacritinib, or momelotinib have shown some activity in RUX pretreated patients [37–39] the outcome after RUX-failure is poor. A large retrospective US population-based study showed for myelofibrosis patients after RUX discontinuation an OS of only 11.1 months [40]. Even if our study suggests better outcome in RUX responders than in those who discontinued RUX because of loss of response the 2-year survival is still about 60% after allografting in patients who failed to RUX. Whether these results can be improved by using a second-line JAK-inhibitor prior to transplant to reduce spleen size after RUX failure has to be shown in a prospective trial. Another concern of postponing transplant by continuing RUX therapy until treatment failure is the observed risk of clonal evolution during RUX treatment by acquiring new mutations [41]. The median duration of RUX in myelofibrosis in the literature is about 3 years but the drug is discontinued due to loss of response or side effects [7, 8, 42].

Our study has some limitations. Due to its retrospective nature, the selection process that decided which patient received RUX prior to stem cell transplantation and which patient was scheduled for transplantation at spleen response or after RUX failure is unknown. It is likely that selection to RUX treatment was based on spleen size which was at a median 12 cm in the RUX pretreatment group and only 8 cm in the non-RUX group. Furthermore, despite the trend for improved outcome for RUX pretreated patients, other factors could drive the results instead of treatment exposure. However, it should be noted that unfavorable factors such as DIPPS intermediate II/high risk and Karnofsky ≤ 80 were more seen in the RUX pretreated group.

In conclusion this large retrospective study showed feasibility of using RUX prior to allogeneic stem cell transplantation in myelofibrosis with no negative impact on NRM, RI, event-free and OS post-transplant. In particular a significant lower graft failure incidence rate was observed in RUX responders in comparison to RUX failure and a significantly lower relapse rate and improved EFS was seen for those who received stem cell transplant with ongoing spleen response to RUX in comparison to non-RUX treated patients.
